# Physical Activity, Functional Performance, and Healthy Aging Across Body Mass Index Categories: A Real-World Primary Care Cross-Sectional Study

**DOI:** 10.3390/life16071107

**Published:** 2026-07-02

**Authors:** Peter Marián Kalanin

**Affiliations:** 1 Institute of General Medicine, Faculty of Medicine, Pavol Jozef Šafárik University in Košice, Rastislavova 45, 040 01 Košice, Slovakia; peter.kalanin@upjs.sk; 2 MED-KAL, s.r.o., Rastislavova 45, 040 01 Košice, Slovakia; 3 Institute of Physical Education and Sport, Pavol Jozef Šafárik University in Košice, Ondavská 21, 040 11 Košice, Slovakia; 4 Department of Physiotherapy, Faculty of Health, Catholic University in Ružomberok, Námestie A. Hlinku 48, 034 01 Ružomberok, Slovakia

**Keywords:** physical activity, BMI, obesity, overweight, LDL cholesterol, functional performance, timed up and go, primary care, healthy aging, functional aging, healthspan, mobility, cardiometabolic risk, cross-sectional study

## Abstract

**Background/Objectives:** Physical activity (PA) and body mass index (BMI) are two central modifiable determinants of cardiometabolic health and functional performance across the adult lifespan. Excess adiposity and physical inactivity frequently coexist and may interact in determining functional aging trajectories. However, evidence from real-world primary care populations examining whether PA–cardiometabolic associations are consistent across BMI categories—including individuals with obesity—remains limited. This study evaluated the associations between self-reported PA categories and LDL-C concentrations and timed up and go (TUG) functional performance across three WHO-defined BMI categories and formally tested whether BMI category modified these associations. **Methods:** This cross-sectional observational study included 863 adult primary care patients stratified into normal weight (BMI < 25 kg/m^2^, *n* = 241), overweight (BMI 25–29.9 kg/m^2^, *n* = 348), and obese (BMI ≥ 30.0 kg/m^2^, *n* = 274) groups. PA was categorized as low, moderate, or high based on World Health Organization recommendations. LDL-C and TUG were the primary outcomes. BMI-category-stratified analyses included one-way ANOVA with Bonferroni post hoc correction and multivariable linear regression adjusted for age, sex, arterial hypertension (AH), and diabetes mellitus (DM). An interaction term (PA × BMI category) formally tested BMI modification. **Results:** Age, LDL-C, TUG, sex, AH, DM, and PA distribution did not differ significantly across BMI categories (all *p* > 0.05). Higher PA categories were associated with significantly lower LDL-C in all three BMI categories (normal: F = 8.93, *p* < 0.001; overweight: F = 10.53, *p* < 0.001; obese: F = 9.35, *p* < 0.001) and with significantly better TUG performance (normal: F = 18.17, *p* < 0.001; overweight: F = 41.62, *p* < 0.001; obese: F = 21.14, *p* < 0.001). PA was the only consistently significant independent predictor of both LDL-C (all *p* < 0.001) and TUG (all *p* < 0.001) in all three BMI categories after multivariable adjustment. The interaction term (PA × BMI category) was not statistically significant for LDL-C (*p* = 0.972) or TUG (*p* = 0.475). The proportion of patients with TUG ≥12 s among low-PA patients increased across BMI categories: 33.7% in patients with normal weight, 37.5% in patients with overweight, and 42.3% in patients with obesity. **Conclusions:** PA–cardiometabolic and PA–functional performance associations were consistent across all three BMI categories, with no statistically significant BMI modification detected. These findings support the potential clinical utility of routine PA assessment as a BMI-independent indicator of cardiometabolic risk and functional health in primary care, including in individuals with obesity, and are relevant to healthy aging and maintaining mobility and functional independence across the adult lifespan.

## 1. Introduction

Physical activity (PA) and body mass index (BMI) are two of the most important modifiable determinants of cardiometabolic health and functional capacity across the adult lifespan [1,2]. Both excess adiposity and physical inactivity are independently associated with adverse lipid profiles, elevated cardiovascular risk, and impaired functional performance—all of which contribute to accelerated functional aging and reduced healthspan [3,4,5]. In primary care settings, BMI and PA are routinely assessed clinical parameters; however, evidence on whether PA–cardiometabolic associations are maintained and consistent across BMI categories—particularly in patients with obesity—remains limited in consecutive primary care cohorts.

Obesity is a highly prevalent and clinically complex condition associated with dyslipidemia, cardiovascular disease, functional impairment, and accelerated functional decline with aging [6,7]. Physical inactivity and excess adiposity frequently coexist, yet their relative and independent contributions to cardiometabolic and functional health outcomes are often examined separately. A clinically important and insufficiently addressed question is whether PA–outcome associations—specifically PA associations with LDL-C concentrations and functional performance—are consistent across BMI categories or whether adiposity significantly modifies these relationships. If PA–outcome associations are BMI-independent, PA assessment may be clinically relevant regardless of a patient’s weight status. If significant BMI modification exists, weight-stratified counseling strategies may be warranted. This question has not been formally examined in a consecutive primary care cohort.

Therefore, the primary aims of the present study were to evaluate BMI-category-stratified associations between self-reported PA categories and LDL-C concentrations and TUG functional performance in a real-world primary care cohort and to formally test whether BMI category modified these associations. We hypothesized that PA would be associated with both outcomes across all BMI categories, with no significant BMI modification.

## 2. Materials and Methods

### 2.1. Study Design and Setting

This study was designed as a cross-sectional observational analysis using routinely collected clinical data from a primary care setting in Slovakia. Data were collected from February 2021 to March 2026. This study was designed and is reported in accordance with the Strengthening the Reporting of Observational Studies in Epidemiology (STROBE) guidelines [8,9]. The present analysis reports BMI-category-stratified findings from the same primary care cohort described in previous publications [10,11,12].

This study was conducted in a real-world solo general practice (MED-KAL, s.r.o., Košice, Slovakia) serving a consecutive, unselected adult population. Patients attended for routine primary care encounters, including periodic preventive check-ups, chronic disease management follow-ups (arterial hypertension, type 2 diabetes mellitus, dyslipidemia), and acute care consultations. Clinical data were collected as part of routine clinical assessment during these visits. The cohort reflects the natural heterogeneity of a consecutive primary care population, which includes patients with varying cardiometabolic comorbidity profiles. The principal comorbidities present in the cohort—arterial hypertension (AH: 55.9% overall) and type 2 diabetes mellitus (DM: 17.3% overall)—are reported in Table 1 and were included as covariates in all multivariable regression models.

### 2.2. Study Population

A total of 863 adult patients aged ≥18 years were included. Inclusion criteria were: age ≥ 18 years, available PA classification, available LDL-C and TUG measurements, and complete core clinical variables. Patients with incomplete records were excluded. No pre-selection was performed based on BMI category, PA level, or cardiometabolic risk profile.

### 2.3. BMI Category Classification

BMI was calculated from routinely recorded weight and height measurements (kg/m^2^). Patients were stratified into three WHO-defined BMI categories: normal weight (BMI < 25.0 kg/m^2^, *n* = 241), overweight (BMI 25.0–29.9 kg/m^2^, *n* = 348), and obese (BMI ≥ 30.0 kg/m^2^, *n* = 274) [13]. Patients with underweight BMI (<18.5 kg/m^2^) were included in the normal weight group due to their small number and were not analyzed separately.

### 2.4. Data Collection

Data were obtained from electronic medical records as part of routine clinical assessment and were anonymized prior to analysis. Clinical variables included age, sex, BMI, LDL-C concentration, AH status, and DM status. AH was defined as a documented clinical diagnosis and/or ongoing antihypertensive treatment. DM was defined as a documented clinical diagnosis and/or ongoing glucose-lowering treatment. LDL-C was measured using an enzymatic colorimetric assay in certified laboratories.

### 2.5. Physical Activity Assessment

PA was assessed using patient self-report during routine clinical evaluation. Based on World Health Organization recommendations, PA was categorized as low (<150 min/week), moderate (150–300 min/week), or high (>300 min/week) [14]. Potential recall bias and exposure misclassification inherent to self-report assessment should be considered when interpreting findings.

### 2.6. Outcome Measures

The primary outcomes were LDL-C concentration (mmol/L) and TUG functional performance (seconds). TUG was assessed using a Garmin digital stopwatch (Garmin Ltd., Olathe, KS, USA) as part of routine primary care evaluation [15]. A TUG time ≥ 12 s was used as an orientational marker of potentially impaired functional mobility for descriptive stratification, acknowledging that this threshold may not apply uniformly across all age groups.

### 2.7. Statistical Analysis

Statistical analyses were conducted using Python (version 3.11) with NumPy and SciPy libraries. Differences in baseline characteristics across BMI categories were assessed using one-way ANOVA for continuous variables and chi-square tests for categorical variables. BMI-category-stratified associations between PA categories and LDL-C and TUG were assessed using one-way ANOVA with Bonferroni post hoc correction. Multivariable linear regression models were constructed separately for each BMI category, with PA entered as an ordinal variable (low = 1, moderate = 2, high = 3). Covariates in the LDL-C model were age, sex, AH, and DM. Covariates in the TUG model additionally included LDL-C. To formally test for BMI modification, an interaction term (PA × BMI category, entered as an ordinal variable: normal = 1, overweight = 2, obese = 3) was added to a pooled model. Statistical significance was set at *p* < 0.05.

### 2.8. Ethical Considerations

This study was conducted in accordance with the Declaration of Helsinki (2013 revision). This study involved secondary analysis of fully anonymized, routinely collected clinical data. According to Slovak legislation governing retrospective anonymized data analysis, formal ethical approval was not required. Patient consent was waived due to the use of anonymized data.

## 3. Results

### 3.1. Baseline Characteristics

The study cohort comprised 241 patients with normal weight (27.9%), 348 overweight (40.3%), and 274 obese (31.7%). Baseline characteristics are presented in Table 1. As expected, BMI differed significantly across groups by design. Age, LDL-C, TUG, sex distribution, AH prevalence, DM prevalence, and PA distribution did not differ significantly across BMI categories (all *p* > 0.05). The proportion of patients with TUG ≥ 12 s showed a non-significant trend toward higher prevalence in the obese group (normal: 19.1%; overweight: 21.6%; obese: 25.9%; *p* = 0.171). This well-balanced cardiometabolic profile across BMI categories allowed for meaningful BMI-stratified comparison of PA–outcome associations.

### 3.2. LDL-C Across PA Categories by BMI Category

Higher PA categories were associated with lower LDL-C across all three BMI categories (all ANOVA *p* < 0.001; Table 2). The mean LDL-C difference between low- and high-PA groups was 0.66 mmol/L in patients with normal weight, 0.60 mmol/L in patients with overweight, and 0.68 mmol/L in patients with obesity—a clinically and statistically consistent gradient across BMI strata. Within each PA category, LDL-C did not differ significantly across BMI categories (all *p* > 0.05).

### 3.3. TUG Performance Across PA Categories by BMI Category

Higher PA categories were similarly associated with better TUG performance across all three BMI categories (all ANOVA *p* < 0.001; Table 3). The mean TUG difference between low- and high-PA groups was 2.07 s in patients with normal weight, 2.45 s in patients with overweight, and 2.31 s in patients with obesity. Notably, the proportion of patients with TUG ≥ 12 s among low-PA patients showed a progressive increase across BMI categories: 33.7% in normal weight, 37.5% in overweight, and 42.3% in obese groups—suggesting that the functional burden of physical inactivity is amplified by increasing adiposity.

### 3.4. Multivariable Regression—LDL-C

Results of BMI category-stratified multivariable regression for LDL-C are presented in Table 4. In all three BMI categories, PA category was the only uniformly significant independent predictor of LDL-C after adjustment for age, sex, AH, and DM: normal weight (unstandardized B = −0.347, 95% CI: −0.504 to −0.190, *p* < 0.001), overweight (B = −0.273, 95% CI: −0.399 to −0.148, *p* < 0.001), and obese (B = −0.335, 95% CI: −0.487 to −0.183, *p* < 0.001). Age, sex, and AH were not independently associated with LDL-C in any BMI category (all *p* > 0.05). DM was significantly associated with LDL-C in the overweight group (B = 0.314, *p* = 0.022) but not in normal weight or obese groups. The overall model R^2^ ranged from 0.078 (normal) to 0.085 (obese). The interaction term (PA × BMI category) was not statistically significant (*p* = 0.972), indicating no significant BMI modification of the PA–LDL-C association.

### 3.5. Multivariable Regression—TUG

Results of BMI-category-stratified multivariable regression for TUG are presented in Table 5. In all three BMI categories, PA category was the only uniformly significant independent predictor of TUG performance after adjustment: normal weight (B = −1.037, 95% CI: −1.397 to −0.677, *p* < 0.001), overweight (B = −1.322, 95% CI: −1.609 to −1.035, *p* < 0.001), and obese (B = −1.159, 95% CI: −1.544 to −0.774, *p* < 0.001). Age showed a weak but statistically significant association with TUG in the normal weight group (B = 0.013, *p* = 0.044) but not in overweight or obese groups. LDL-C, sex, AH, and DM were not independently associated with TUG in any BMI category (all *p* > 0.05). The overall model R^2^ was 0.148 (normal), 0.207 (overweight), and 0.139 (obese). The interaction term (PA × BMI category) was not statistically significant (*p* = 0.475), indicating no significant BMI modification of PA–TUG association.

## 4. Discussion

### 4.1. Principal Findings

In this real-world primary care cohort of 863 adults stratified into three WHO-defined BMI categories, higher self-reported PA categories were associated with lower LDL-C concentrations and better TUG functional performance across all three BMI groups. PA was the only consistently significant independent predictor of both outcomes in all three BMI categories after multivariable adjustment. Formal interaction testing indicated no significant BMI modification of either the PA–LDL-C (*p* = 0.972) or PA–TUG association (*p* = 0.475), indicating that PA–health associations were BMI-independent in this primary care population. This observation is consistent with the concept that physical activity may partially offset the functional consequences of excess adiposity across the adult lifespan [16].

A clinically important observation is the progressive increase in the TUG ≥12 s prevalence among low-PA patients across BMI categories: 33.7% in the normal weight, 37.5% in the overweight, and 42.3% in the obese group. While this gradient was not statistically significant at the group level (*p* = 0.171), it suggests that the functional burden of physical inactivity is amplified by increasing adiposity, consistent with the known interactive effects of obesity and inactivity on functional performance. This pattern highlights the particular importance of PA assessment and counseling in patients with obesity in primary care.

The finding that the regression coefficient for PA on TUG was largest in the overweight group (B = −1.322) compared with the normal weight (B = −1.037) and obese (B = −1.159) groups, while not reaching significance in formal interaction testing, suggests a modest BMI-related gradient in the PA–functional performance association. However, this difference should be interpreted cautiously given the absence of a significant interaction. Although causal inference cannot be established from cross-sectional data, the remarkably similar effect sizes across BMI categories support the robustness and potential clinical relevance of the observed associations.

### 4.2. Comparison with the Existing Literature

The present findings are broadly consistent with existing evidence demonstrating associations between PA and lipid profiles across BMI categories [1,3,17,18]. Previous studies have demonstrated that exercise-related improvements in lipid profiles occur across weight categories, including in patients with obesity, though the magnitude of benefit may vary [4,19,20]. The present cross-sectional findings extend this evidence to a consecutive real-world primary care population, demonstrating consistent PA–LDL-C associations with similar magnitudes across BMI strata (LDL-C differences of 0.60–0.68 mmol/L between low- and high-PA groups).

Regarding functional performance, the literature consistently supports that PA is a stronger determinant of functional capacity than BMI alone, with patients with obesity who are physically active demonstrating better functional outcomes than sedentary individuals with normal weight [21,22]. The present findings align with this evidence, showing that PA category explained the similar proportions of TUG variance across BMI categories (R^2^ = 0.139–0.207) and that PA was the dominant predictor of TUG in all three groups, independent of adiposity.

### 4.3. Clinical and Public Health Implications

The consistency of PA–outcome associations across BMI categories supports the potential clinical utility of routine PA assessment as a BMI-independent component of cardiometabolic and functional health evaluation in primary care. These findings suggest that PA screening and counseling should not be de-prioritized in patients with overweight or obesity on the assumption that weight management alone will suffice for cardiometabolic and functional risk reduction. Rather, PA and BMI may represent independent and complementary clinical targets.

The progressive increase in functional impairment among low-PA patients across BMI categories further underscores the importance of integrated assessment of both PA and BMI in primary care. Routine PA screening may help identify patients at elevated functional risk within each BMI stratum, regardless of weight status—a finding with direct relevance to healthy aging and the preservation of functional independence in primary care populations. This finding is consistent with the broader series of real-world analyses from this cohort supporting PA assessment as a practical, low-cost screening tool across diverse primary care patient profiles.

The present findings have direct implications for patient-centered PA counseling in primary care. A recent qualitative systematic review by Christensen et al. [23] examined the desires and perspectives of individuals with overweight and obesity regarding physical activity participation, identifying key psychological, social, and environmental barriers to PA engagement in this population and highlighting the importance of patient-centered approaches to PA promotion. The quantitative evidence from the present study—demonstrating that PA benefitting LDL-C concentrations and TUG functional performance are consistent across BMI categories—provides a complementary empirical rationale for initiating PA screening and counseling without delay in overweight and obese primary care patients, rather than deferring such interventions until weight loss targets are achieved. Integrating patient-centered perspectives on PA participation, as described by Christensen et al. [23], with evidence for BMI-independent PA benefits may support the development of more effective and inclusive PA promotion programs in primary care. Notably, sex-stratified analyses from the present cohort similarly demonstrated that PA–LDL-C and PA–TUG associations were consistent across males and females, further supporting the generalizability of PA assessment as a universal primary care tool [24].

### 4.4. Limitations

Several limitations should be acknowledged. First, the cross-sectional design precludes causal inference and reverse causation cannot be excluded. Second, PA was assessed using self-report without a validated questionnaire or objective monitoring, introducing potential recall bias. Third, BMI is a crude and imperfect measure of adiposity that does not distinguish between fat mass and lean mass, and does not capture body fat distribution. Visceral obesity—which is increasingly recognized as a key driver of cardiovascular and metabolic pathology independent of total body weight [25]—cannot be assessed from BMI alone. Future studies should incorporate waist circumference, waist-to-hip ratio, or body composition measurement (e.g., dual-energy X-ray absorptiometry, bioelectrical impedance) to more precisely characterize adiposity phenotypes and the effects of their potential interaction with physical activity on cardiometabolic and functional outcomes. Fourth, although regression models were adjusted for age, sex, AH, and DM, residual confounding from unmeasured variables—including dietary habits, musculoskeletal conditions, and socioeconomic factors—cannot be excluded. Fifth, information regarding lipid-lowering therapy (e.g., statins, ezetimibe) was not available for adjustment, as these data were not systematically recorded as structured variables in the electronic medical records used for this retrospective analysis. Statin use may independently lower LDL-C concentrations and could represent a confounding factor if differentially distributed across PA categories. Notably, if patients with known dyslipidemia are more likely to be both physically active (motivated by awareness of their cardiometabolic risk) and to receive lipid-lowering pharmacotherapy, any confounding effect would tend to attenuate rather than inflate the observed PA–LDL-C association, suggesting the true independent PA effect is at least as large as reported. Future studies with structured pharmacotherapy data are needed to disentangle independent PA effects on LDL-C from concurrent lipid-lowering treatment. Similarly, the absence of dyslipidemia as a formally documented clinical diagnosis (as a distinct structured variable separate from measured LDL-C) represents a further limitation. Sixth, this study was conducted in a single primary care practice in Slovakia, which may limit generalizability. This study was conducted by a single primary care physician in a real-world outpatient setting, which inherently reflects the single-practitioner nature of primary care practice in Slovakia. Residual confounding therefore cannot be completely excluded despite multivariable adjustment.

### 4.5. Future Directions

Future studies should incorporate objective PA assessment (accelerometry) and body composition measurement to more precisely characterize the effects of PA–adiposity interactions on cardiometabolic and functional outcomes across the aging continuum. Longitudinal designs would allow evaluation of whether PA-associated reductions in LDL-C and improvements in TUG performance are maintained across BMI trajectories over time—a question of particular relevance for healthy aging research. Studies including frailty assessment, body composition data, and longitudinal tracking of functional performance would provide more complete insight into the role of PA in preserving functional independence across BMI categories in aging primary care populations. Future studies should also evaluate frailty trajectories and longitudinal changes in functional independence as primary outcomes, using established frailty instruments to characterize the intersection of physical inactivity, adiposity, and functional aging.

## 5. Conclusions

In this real-world primary care cohort, higher self-reported PA was associated with lower LDL-C concentrations and better TUG functional performance across all three WHO-defined BMI categories—normal weight, overweight, and obese. PA category was the only consistently significant independent predictor of both outcomes in all BMI categories after multivariable adjustment, with no statistically significant BMI modification detected. A progressive increase in functional impairment among low-PA patients across BMI categories suggests that the functional burden of inactivity is amplified by increasing adiposity. These findings support routine physical activity assessment as a practical, BMI-independent component of cardiovascular and functional risk evaluation in primary care, including in individuals with obesity, and are relevant to healthy aging and the maintenance of mobility and functional independence across the adult lifespan.

## Figures and Tables

**Table 1:** Baseline demographic and clinical characteristics by BMI category. Continuous variables compared using one-way ANOVA. Categorical variables compared using chi-square test. * BMI differs by group design. Abbreviations: AH, arterial hypertension; BMI, body mass index; DM, diabetes mellitus; LDL-C, low-density lipoprotein cholesterol; PA, physical activity; SD, standard deviation; TUG, timed up and go.

Variable	Normal Weight BMI < 25 (*n* = 241)	Overweight BMI 25–29.9 (*n* = 348)	Obese BMI ≥ 30 (*n* = 274)	*p*
Age (years), mean ± SD	50.7 ± 19.6	50.4 ± 19.5	51.6 ± 18.8	0.752
BMI (kg/m^2^), mean ± SD	22.1 ± 2.4	27.4 ± 1.4	32.8 ± 2.5	<0.001 *
Female sex, *n* (%)	120 (49.8%)	174 (50.0%)	145 (52.9%)	0.713
LDL-C (mmol/L), mean ± SD	3.55 ± 0.93	3.52 ± 0.94	3.57 ± 0.94	0.804
TUG (seconds), mean ± SD	10.23 ± 2.13	10.16 ± 2.25	10.22 ± 2.37	0.915
TUG ≥ 12 s, *n* (%)	46 (19.1%)	75 (21.6%)	71 (25.9%)	0.171
AH, *n* (%)	143 (59.3%)	186 (53.4%)	152 (55.5%)	0.366
DM, *n* (%)	44 (18.3%)	52 (14.9%)	52 (19.0%)	0.360
Low PA, *n* (%)	95 (39.4%)	144 (41.4%)	104 (38.0%)	0.401
Moderate PA, *n* (%)	100 (41.5%)	131 (37.6%)	124 (45.3%)	
High PA, *n* (%)	46 (19.1%)	73 (21.0%)	46 (16.8%)	

**Table 2:** LDL-C concentrations across PA categories by BMI category. Post hoc comparisons performed using Bonferroni correction. Abbreviations: BMI, body mass index; LDL-C, low-density lipoprotein cholesterol; PA, physical activity; SD, standard deviation.

PA Category	Normal Weight LDL-C Mean ± SD	Overweight LDL-C Mean ± SD	Obese LDL-C Mean ± SD	ANOVA *p* Within Group
Low PA	3.82 ± 0.95 (*n* = 95)	3.70 ± 0.97 (*n* = 144)	3.83 ± 0.96 (*n* = 104)	
Moderate PA	3.47 ± 0.93 (*n* = 100)	3.56 ± 0.93 (*n* = 131)	3.50 ± 0.89 (*n* = 124)	
High PA	3.16 ± 0.71 (*n* = 46)	3.10 ± 0.74 (*n* = 73)	3.15 ± 0.85 (*n* = 46)	
ANOVA *p*	*p* < 0.001 (F = 8.93)	*p* < 0.001 (F = 10.53)	*p* < 0.001 (F = 9.35)	All *p* < 0.001

**Table 3:** TUG performance across PA categories by BMI category. Post hoc comparisons performed using Bonferroni correction. Abbreviations: BMI, body mass index; PA, physical activity; SD, standard deviation; TUG, Timed Up and Go.

PA Category	Normal Weight TUG Mean ± SD (s)	Overweight TUG Mean ± SD (s)	Obese TUG Mean ± SD (s)	ANOVA *p* Within Group
Low PA	11.09 ± 2.19 (*n* = 95)	11.29 ± 2.25 (*n* = 144)	11.26 ± 2.38 (*n* = 104)	
Moderate PA	9.98 ± 2.03 (*n* = 100)	9.66 ± 2.02 (*n* = 131)	9.82 ± 2.27 (*n* = 124)	
High PA	9.02 ± 1.40 (*n* = 46)	8.84 ± 1.50 (*n* = 73)	8.95 ± 1.58 (*n* = 46)	
ANOVA *p*	*p* < 0.001 (F = 18.17)	*p* < 0.001 (F = 41.62)	*p* < 0.001 (F = 21.14)	All *p* < 0.001

**Table 4:** BMI-category-stratified multivariable linear regression for LDL-C. PA entered as ordinal variable (low = 1, moderate = 2, high = 3). * *p* < 0.05; *** *p* < 0.001. Abbreviations: AH, arterial hypertension; BMI, body mass index; CI, confidence interval; DM, diabetes mellitus; LDL-C, low-density lipoprotein cholesterol; PA, physical activity.

Variable	Normal B	Normal 95% CI	Overweight B	Overweight 95% CI	Obese B	Obese 95% CI
PA (per increase) ***	−0.347	−0.504 to −0.190	−0.273 ***	−0.399 to −0.148	−0.335 ***	−0.487 to −0.183
Age (years)	0.000	−0.006 to 0.006	−0.004	−0.009 to 0.001	−0.002	−0.007 to 0.004
Sex (male)	0.082	−0.151 to 0.315	0.036	−0.157 to 0.228	−0.158	−0.375 to 0.058
AH	−0.003	−0.237 to 0.231	0.160	−0.033 to 0.352	−0.077	−0.295 to 0.140
DM	−0.204	−0.502 to 0.093	0.314 *	0.046 to 0.583	−0.277	−0.553 to 0.000
R^2^	0.078		0.079		0.085	
Interaction PA × BMI *p* (LDL)	*p* = 0.972					

**Table 5:** BMI-category-stratified multivariable linear regression for TUG. PA entered as ordinal variable. * *p* < 0.05; *** *p* < 0.001. Abbreviations: AH, arterial hypertension; BMI, body mass index; CI, confidence interval; DM, diabetes mellitus; LDL-C, low-density lipoprotein cholesterol; PA, physical activity; TUG, Timed Up and Go.

Variable	Normal B	Normal 95% CI	Overweight B	Overweight 95% CI	Obese B	Obese 95% CI
PA (per increase) ***	−1.037	−1.397 to −0.677	−1.322 ***	−1.609 to −1.035	−1.159 ***	−1.544 to −0.774
Age (years)	0.013 *	0.000 to 0.026	0.010	−0.001 to 0.021	0.000	−0.014 to 0.014
LDL-C (mmol/L)	0.032	−0.250 to 0.314	−0.161	−0.397 to 0.076	0.183	−0.110 to 0.477
Sex (male)	0.068	−0.447 to 0.582	−0.073	−0.502 to 0.357	−0.110	−0.642 to 0.423
AH	0.096	−0.420 to 0.612	0.131	−0.299 to 0.560	0.065	−0.469 to 0.599
DM	0.051	−0.608 to 0.709	0.532	−0.071 to 1.135	0.320	−0.364 to 1.003
R^2^	0.148		0.207		0.139	
Interaction PA × BMI *p* (TUG)	*p* = 0.475					

## Data Availability

The datasets used and/or analyzed during the current study are available from the corresponding author on reasonable request.
